# A biophysical vascular bubble model for devising decompression procedures

**DOI:** 10.14814/phy2.13191

**Published:** 2017-03-21

**Authors:** Ran Arieli, Abraham Marmur

**Affiliations:** ^1^ Israel Naval Medical Institute, Haifa, and Eliachar Research LaboratoryWestern Galilee Medical CenterNahariyaIsrael; ^2^ Department of Chemical EngineeringTechnion‐Israel Institute of TechnologyHaifaIsrael

**Keywords:** Active hydrophobic spot, arterial bubbles, bubble expansion, decompression illness

## Abstract

Vascular bubble models, which present a realistic biophysical approach, hold great promise for devising suitable diver decompression procedures. Nanobubbles were found to nucleate on a flat hydrophobic surface, expanding to form bubbles after decompression. Such active hydrophobic spots (AHS) were formed from lung surfactants on the luminal aspect of ovine blood vessels. Many of the phenomena observed in these bubbling vessels correlated with those known to occur in diving. On the basis of our previous studies, which proposed a new model for the formation of arterial bubbles, we now suggest the biophysical model presented herein. There are two phases of bubble expansion after decompression. The first is an extended initiation phase, during which nanobubbles are transformed into gas micronuclei and begin to expand. The second, shorter phase is one of simple diffusion‐driven growth, the inert gas tension in the blood remaining almost constant during bubble expansion. Detachment of the bubble occurs when its buoyancy exceeds the intermembrane force. Three mechanisms underlying the appearance of arterial bubbles should be considered: patent foramen ovale, intrapulmonary arteriovenous anastomoses, and the evolution of bubbles in the distal arteries with preference for the spinal cord. Other parameters that may be quantified include age, acclimation, distribution of bubble volume, AHS, individual sensitivity, and frequency of bubble formation. We believe that the vascular bubble model we propose adheres more closely to proven physiological processes. Its predictability may therefore be higher than other models, with appropriate adjustments for decompression illness (DCI) data.

## Introduction

Various physiological models have been used to design safe procedures for diver decompression. In the construction of decompression tables, the models followed in common diving practice assumed different types of tissue having variable thresholds, the elimination of inert gas both in solution and in gaseous form, and different definitions of decompression risk. These assumptions are many and varied, and large numbers of parameters were used to fit the models to the actual decompression risk. One model in common use is the “reduced gradient bubble model” of Wienke, who described dive modeling as “often more of an artform than science” (Wienke 2009). Hugon (2014) recently summarized the various approaches to decompression, suggesting that “vascular bubble models, proposing a realistic biophysical approach, are promising for the prevention of decompression sickness and the devising of suitable decompression procedures.” Vascular bubbles, and arterial bubbles in particular, are the cause of most cases of severe decompression illness (DCI) (Eftedal et al. 2007). The inflammation, neutrophil activation, and platelet aggregation seen in DCI are due to microparticles composed of cell membranes, which we and others believe to have been stripped from the vasculature by bubbles (Arieli et al. 2015; Pontier et al. 2009b; Yang et al. 2012). Support for this assumption is discussed further in the section below on “bubble expansion and detachment” (*Detachment*, points i–vii). It has even been suggested that cutis marmorata, generally considered to be a relatively mild form of DCI, is related to vascular bubbles in the medulla oblongata (Germonpre et al. 2015; Jitsuiki et al. 2015). We therefore suggest that most symptoms of DCI aside from joint pain and lymphatic swelling, and perhaps also bubble formation in the myelin of the spinal cord (Francis et al. 1990), are related to vascular bubbles. Decompression bubbles in the joints can be related to a cavitation mechanism (Yanagawa et al. 2016) and may be modeled separately. The main tissue with which we should be concerned when considering decompression and DCI is therefore the vascular compartment.

We recently suggested a new mechanism of bubble nucleation. Nanobubbles are formed on a flat hydrophobic surface (silicon wafers) from dissolved gas (Tyrrell and Attard 2001; Yang et al. 2007). It was demonstrated that after decompression, these nanobubbles expand to form bubbles (Arieli and Marmur 2011, 2013a). We further showed that there are active hydrophobic spots (AHS), which stained for lipids and produced bubbles after decompression, on the luminal aspect of ovine blood vessels: the aorta, superior vena cava, right and left atria, pulmonary artery and pulmonary vein (Arieli et al. 2015; Arieli and Marmur 2013b, 2014, 2016). These AHS correspond to the oligolamellar phospholipids described by Hills (1992) in various blood vessels in the sheep, including the cerebral capillaries. In our experiments, blood vessels were separated anaerobically under saline, stretched on glass microscope slides, exposed to hyperbaric pressure, and photographed after decompression. We determined, under a pulsatile flow regime, the size of bubbles on detachment, their rate of expansion, and the variability between sheep and within the AHS.

Many of the phenomena we observed in these bubbling vessels correlated with those known to occur in diving (Arieli et al. 2015): variability in total bubble production correlated with bubblers/nonbubblers, stripping of phospholipids by bubbles with adaptation to diving, and activation of the AHS with the increased risk of DCI in a second dive on the same day. The fact that AHS were found in both arterial and venous blood vessels suggests the possibility that bubbles may form in the arterial circulation, which would explain cases of neurological DCI in the absence of a left‐to‐right shunt. We observed two different rates of expansion in the development of decompression bubbles, showing that bubble expansion is composed of two phases: initiation of the AHS, followed by diffusion‐driven expansion (Arieli and Marmur 2016). This makes it possible to eliminate the artificially slow diffusion constants introduced by other models. In the words of Hugon (2014), “Physical artifacts such as very low diffusion coefficients were sometimes introduced to slow down the bubble growth process, in order to echo the observed DCS symptom delay. This assumption remains questionable.”

On the basis of our previous studies, which proposed a new model for the formation of arterial bubbles, we now suggest a biophysical mechanism and the distribution of its components to enable the construction of reliable, scientifically based decompression procedures. A two‐phase mechanism of bubble expansion and detachment from the blood vessel is suggested. Venous bubbles might block lung perfusion, but arterial bubbles could have their origin in a patent foramen ovale (PFO), intrapulmonary arteriovenous anastomoses (IPAVA), and the possible evolution of bubbles in the arterial circulation as suggested here. The variability in distribution, activity, and activation of the AHS between animals is presented, as are the differences within the AHS themselves.

## Methods

Our analysis is based on experimental work carried out in our laboratory and described in a number of previous publications (Arieli et al. 2015, 2016; Arieli and Marmur 2011, 2013a,b, 2014, 2016). The research method using sheep blood vessels is described briefly here. The complete heart and lungs from slaughtered sheep were obtained at the abattoir. In the laboratory, under saline and without any exposure to air, samples from four blood vessels, the aorta, superior vena cava, pulmonary vein and pulmonary artery, were gently stretched on microscope slides using metal clips with the luminal aspect exposed. Slides were placed anaerobically on the bottom of a Pyrex bowl. The bowl was placed in the cooled high‐pressure chamber (1000 kPa) for about 20 h and photographed after decompression. A pulsatile flow of saline delivered over the blood vessel mimicked arterial flow. It was possible to determine active hydrophobic spots by observing the formation of bubbles at specific locations. The theoretical analysis of these arterial bubbles is original to the present report.

### Bubble expansion and detachment

In our recent study (Arieli and Marmur 2016), we showed that there are two phases in the expansion of bubbles formed on the luminal aspect of blood vessels after decompression.

#### Phase I

**Figure 1:**
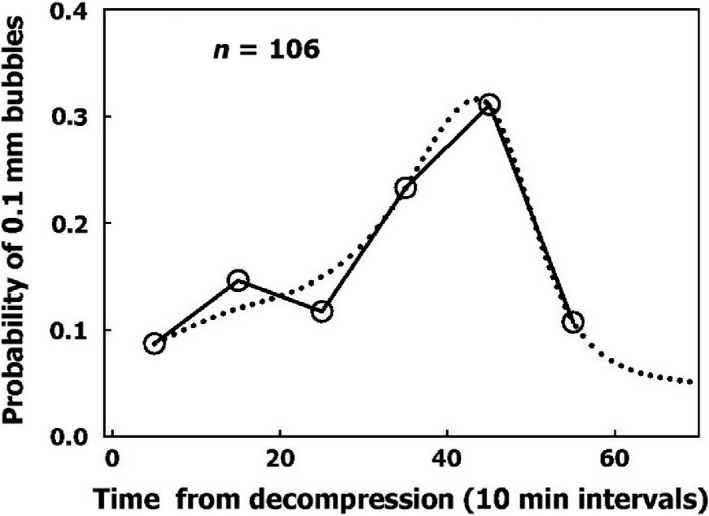
Frequency of initiation of active hydrophobic spots (AHS). Initiation is defined as the moment the first bubble from an AHS reaches a diameter of 0.1 mm as a function of time from decompression. The dotted line is a suggested smooth function.

This is an extended phase, in which nanobubbles are transformed into gas micronuclei and begin to expand. We termed this phase “initiation of the AHS.” Its time scale can be seen in Figure 1, which shows when the first bubble from each AHS reached a diameter of 0.1 mm (our lowest photographic resolution). These data have been extracted from our previous studies (Arieli et al. 2015; Arieli and Marmur 2016). It may be seen that the initiation phase for an AHS could take at least 1 h from decompression to reach completion, peaking at 45 min. Because measurements were terminated 1 h after decompression, a smooth dashed curve describes our suggested function. Variability in initiation of the AHS may reflect competition for gas between nanobubbles, the distribution of size, shape, and radius of their curvature, or variability between the various AHS.

#### Phase II

We have shown that the increase in diameter from 0.1 mm until a bubble detaches from the blood vessel is linear with time (Arieli and Marmur 2014, 2016). This linear expansion of the diameter is in agreement with the assumption that gas exchange during this phase is driven by simple diffusion alone, with the inert gas tension in the blood remaining constant during the expansion of a single bubble until detachment (Appendix to Arieli and Marmur 2014).

In our experiments, the diameter of the bubbles increased linearly with time, suggesting that the tension of the inert gas in the flowing saline was constant. In 2–24 min, it took for a bubble with a diameter of 0.1 mm to reach the size at which it became detached from the blood vessel (Arieli and Marmur 2016), the change in saline gas tension was negligible. Similarly, in decompression from a dive, the inert gas tension in the venous blood, which drains all of the tissues, may be considered to remain constant over the short period taken by bubbles to develop and become detached. Thus, gas transfer by diffusion alone may be used for the second phase in our modeling of decompression procedures.

#### Detachment

Bubbles detached from the hydrophobic surface of silicon wafers when they reached a diameter of 4.2 mm, which can be expressed as a buoyancy force of 38 × 10^−6^ N. This is the expected force on detachment for hydrophobicity with a contact angle of 90° (Arieli and Marmur 2014). Bubbles became detached from the AHS on the luminal aspect of blood vessels at a smaller diameter, a mean of 1.0 mm, which is equivalent to 4.5 × 10^−6^ N. Pulsatile saline flow at normal blood flow velocity did not cause the detachment of bubbles any smaller than those which became detached in calm conditions (Arieli et al. 2015). Therefore, buoyancy is the main force inducing bubble detachment.

There are two possible explanations for the detachment of bubbles at a smaller volume from blood vessels compared with hydrophobic silicon wafers. Either the AHS are small and irregular, having a perimeter that enables only limited contact between bubble and tissue, or it may be that the underlying phospholipids become detached along with the bubble. A number of findings serve as evidence in support of the second assumption. (1) Bubbles that detach from AHS having a large surface area, which produce a few bubbles at a time, are no larger than others on detachment (Arieli and Marmur 2016). (2) Subsequent staining for lipids failed to show most of the AHS which produced only one or two bubbles. The explanation for this was that phospholipids were carried away from the AHS along with the bubbles, which correlates with adaptation to diving (Arieli et al. 2015). (3) After diving, there are endothelial microparticles in the blood which are composed of stripped endothelial membranes (Thom et al. 2011). (4) Some of these enlarged microparticles contain gas (Yang et al. 2012). (5) Diving caused a reduction in endothelial function (Madden et al. 2010; Obad et al. 2007), which points to a damaged endothelium. (6) Endothelial damage (functional and anatomical) due to decompression bubbles was demonstrated in the pulmonary artery of the pig (Nossum et al. 1999) and rat (Zhang et al. 2016). (7) The point of contact between the membraneous bilayer is prone to cavitate in clinically used ultrasound (Krasovitski et al. 2011), which suggests a weak adhesion force.

**Figure 2:**
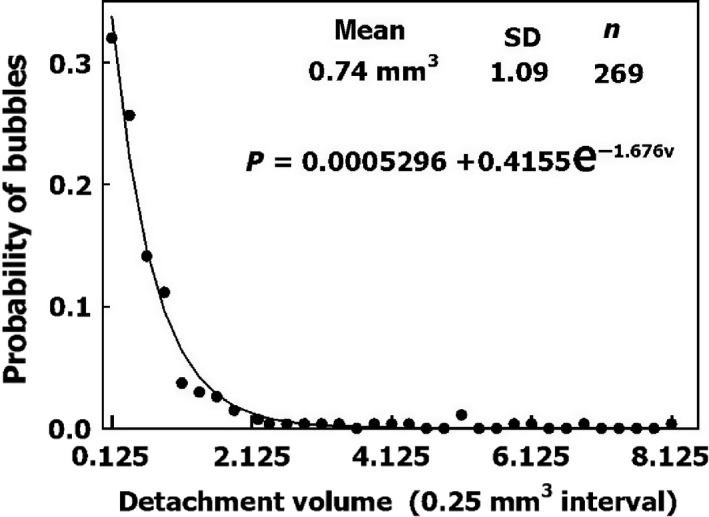
Distribution of bubble volume on detachment under pulsatile flow. An exponential function was used to fit the data.

The distribution of bubble volume on detachment is shown in Figure 2. Very few bubbles detached with a diameter of <0.6 mm, and almost no bubbles detached with a diameter of <0.4 mm. The volumes can be expressed as force at detachment by calculating the buoyancy of the bubble, 1 mm^3^ being equivalent to 9.80 × 10^−7^ N.

### Arterial bubbles

Whenever there is no known specific risk factor for decompression illness, the distribution of risk factors in divers needs to be considered. The risk of a right‐to‐left shunt of venous bubbles related to PFO has been investigated in a number of studies (Billinger et al. 2011; Germonpré et al. 1998; Torti et al. 2004; Wilmshurst et al. 2015). Others discuss the risk of venous arterial shunts due to IPAVA and exercise (Ljubkovic et al. 2012; Madden et al. 2013). However, replacing the traditional dive profile of descent, followed by bottom time and ascent, with repeated ascents and descents during a dive, as practiced for example in “yo‐yo” diving, should raise suspicion of an added risk if we take into consideration the possibility of IPAVA.

Because AHS are to be found in blood vessels within the arterial as well as the venous circulation, with a distribution going as far as the cerebral capillaries (Hills 1992), one ought to consider the possibility that bubbles develop within the arteries. This mechanism can explain symptoms of neurological decompression sickness that occur without arterialization of venous blood, either via a PFO or IPAVA (Madden et al. 2015). After decompression, inert gas is released from the pulmonary capillaries to the lung. However, at any bifurcation, flow decreases for each vessel with the distance from the heart along the arterial tree. The vessel's diameter is reduced, which increases the surface area available for diffusion with respect to blood volume, and the reduced wall thickness reduces the diffusion barrier. Thus, the diffusion of inert gas from the tissue into the blood will rise along the arterial tree. This may cause the expansion of bubbles at AHS within the arteries.

**Figure 3:**
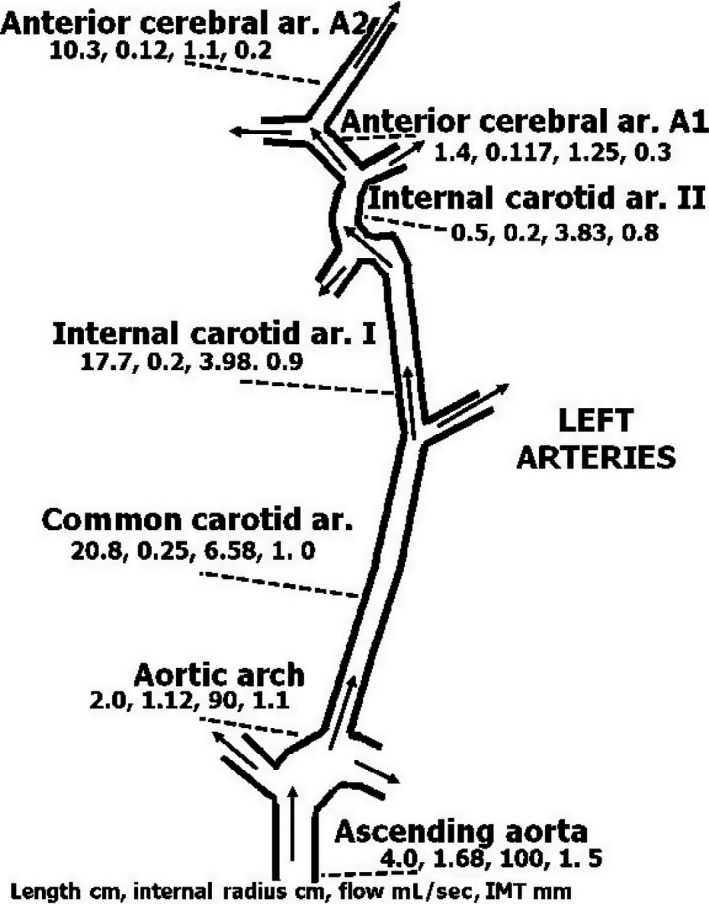
Diagram of specific left arterial blood supply to the brain from the aorta to the anterior cerebral artery. Name of the artery, length (cm), internal diameter (cm), blood flow (mL/sec), and wall thickness (mm) are given from left to right below each. Wall thickness refers to intima‐media thickness (IMT). Because the adventitia is rich in blood supply, it is included with the well‐mixed surrounding tissue. References for sources of the data are provided in the text.

An example of an arterial path is shown in Figure 3: the aorta, leading to the common carotid artery, internal carotid artery, and anterior cerebral artery. The data were compiled from different sources (Ackroyd et al. 1986; Alastruey et al. 2007; Bogren et al. 1994; Enzmann et al. 1994; Mao et al. 2008; Silvestrini et al. 2002; Tokuda et al. 2008 and others). The dimensions given for each artery are (reading from left to right): length (cm), internal radius (cm), blood flow (mL/sec), and wall thickness (mm). Wall thickness is considered a diffusion barrier between well‐mixed tissue and well‐mixed blood, and is composed of the two inner layers of the artery, the intima and the media. The outer layer, the adventitia, which is rich in blood supply, is therefore assigned to the well‐mixed tissue.

**Figure 4:**
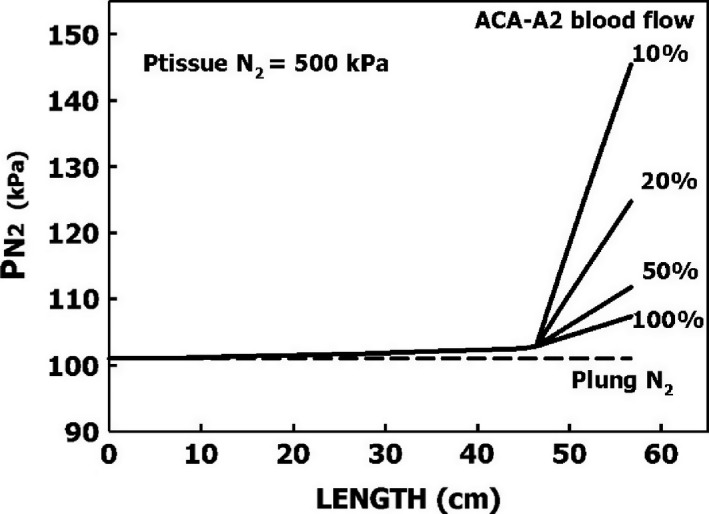
Calculated inert gas tensions (Appendix) along the arterial tree, as depicted in Figure 3, at steady state when gas tension in the surrounding tissue is 500 kPa and in the left ventricle 101 kPa. Values are also calculated for reduction of blood flow in the anterior cerebral artery (A2) to 50%, 20%, and 10%.

Inert gas tension along the length of the arterial segment was calculated using the equation (Appendix):

$$\text{Px}={\text{PT‐(PT‐P0)e}}^{(-2\pi D/\mathrm{WV})x}$$

where Px, PT, and P0 are the gas tensions at distance x from the entrance to the vessel, in the surrounding tissue, and at the entrance to the vessel, respectively; *r* is the internal radius, W is wall thickness, V is blood flow, D is the diffusion coefficient, and x is the distance along the length of the vessel. We used this equation to calculate gas tension along the length of the arterial tree in Figure 3 for a PT of 500 kPa and a P0 of 101 kPa at the entrance to the aorta, as shown in Figure 4. The rate of inert gas loading is seen to increase with the reduction in vessel diameter, wall thickness, and blood flow. Gas tension increased in the present example by 1% at the end of the anterior cerebral artery. However, when blood flow in the anterior cerebral artery (A2) is reduced to 10%, gas tension increases by 44%. Further down along the arterial tree from the anterior cerebral artery, the reduction in flow and decrease in wall thickness may cause an increase in inert gas tension that will result in bubble expansion. If a bubble causes a reduction in flow, it will enhance the entry of inert gas into the vessel. The first phase in arterial bubble growth (the initiation phase) could start during decompression, when the arterial blood is loaded with inert gas. The increase of about 7% in cerebral perfusion within 30 min of decompression (Barak et al. 2016) may protect the brain from the development of arterial bubbles.

Imbert et al. (2004) also suggested that bubble expansion from the large amount of stored nitrogen took place within the brain, but their hypothesized source was tiny bubbles which had passed through the lung capillaries during decompression and not local AHS. In our experiments, the detachment of bubbles with a diameter of <0.4 mm was a very rare event. Therefore, tiny bubbles at the AHS would not be expected to detach and pass through the lung capillaries during decompression. Expansion of bubbles downstream in the arterial tree may explain the microvascular insult and the focal, highly localized punctate lesions seen in the white matter of experienced divers (Connolly and Lee 2015). Deterioration of neuropsychometric performance was found in experienced divers compared with nondiver controls (Balestra and Germonpré 2016). However, there was no difference in performance between divers with and without a PFO, so that this may also be related to bubbles which develop in the more remote branches of the arterial tree, and not to bubbles shunting from the right to the left heart. Schipke and Tetzlaff (Schipke and Tetzlaff 2016) recently related the predominance of neurological decompression sickness in breath‐hold divers to shunting via IPAVA due to the development of hypoxia. In line with some of the responses to their article which proposed alternative mechanisms, we may suggest that because the arterial blood during a breath‐hold dive is rich in dissolved nitrogen, bubble expansion within the arteries of the central nervous system may rather be the correct explanation.

Because the brain receives eight times the amount of arterial blood directed to the spinal cord, more arterial bubbles should reach the brain (Hallenbeck et al. 1975). However, spinal DCI is over three times more frequent than cerebral DCI. The internal veins which drain the spinal cord do not have valves (Stringer et al. 2012). It was also suggested that after decompression, spinal blood flow is reduced due to obstructions in the epidural vertebral veins (Hallenbeck et al. 1975). It is therefore possible that whereas cerebral circulation following a dive may increase (Havnes et al. 2013), the reduction in spinal arterial blood flow could enhance the formation of arterial bubbles in the spinal cord (Fig. 4). This might account for the increased risk of spinal rather than cerebral DCI.

In summary, three mechanisms of arterial bubbles should be considered: PFO, IPAVA, and the evolution of bubbles in the distal arteries with preference for the spinal cord.

### Variability between sheep and variability between active hydrophobic spots

#### Individual sensitivity to DCS

**Figure 5:**
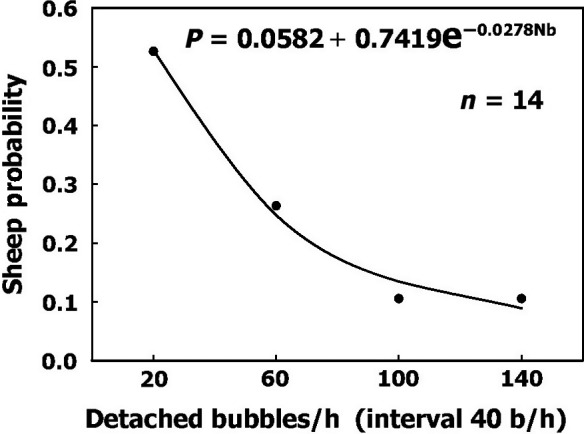
Frequency of sheep having various levels of bubble production after decompression. An exponential function was used to fit the data.

It is a prerequisite for the development of decompression procedures that we take into account the variability between divers and within physiological parameters. Variability between divers (bubblers/nonbubblers) is similar to the variability between sheep depicted in Figure 5. About half of the sheep were low bubble producers, and the frequency of high bubble‐producing sheep declined exponentially with the increase in bubble production. A suggested exponential equation for the probability of bubble production per hour in a sheep is:

P = 0.0582 + 0.7419e^−0.0278×bubbles/h^

This equation can be used to express different levels of sensitivity in divers on a scale running from nonbubblers to heavy bubblers.

**Figure 6:**
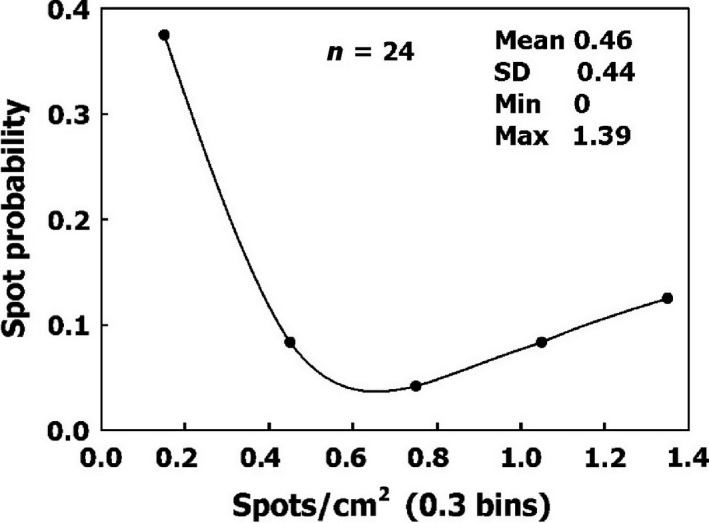
Frequency of AHS density on blood vessels.

There was no difference in the distribution of AHS in the different blood vessels. All of the parameters for the AHS from the four blood vessels and for all sheep were therefore combined. The densities of AHS for the four blood vessels and six sheep were divided into six bins, and their probability is shown in Figure 6. It may be assumed that this characteristic of AHS density will prevail in any blood vessel. The smooth line describing the probability of different levels of AHS density can be used for quantification.

#### AHS bubble production

**Figure 7:**
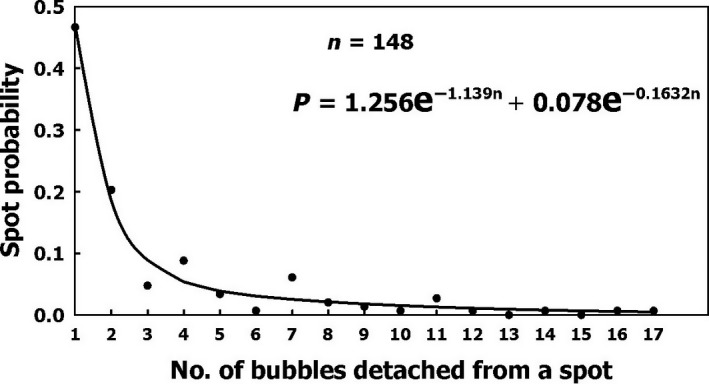
Frequency of AHS as a function of their productivity: the number of bubbles which became detached within 1 h. Two exponential functions were found which could delineate the frequency.

Bubble production varied between the different AHS. The frequency of AHS according to their productivity is shown in Figure 7. The number of AHS decreases as bubble productivity increases. A second exponential equation provides a good description of the probability of bubble production by an AHS within 1 h:

P = 1.256e^−1.139n^ + 0.078e^−0.1632n^ where n is the number of bubbles produced within 1 h.

#### Activation

**Figure 8:**
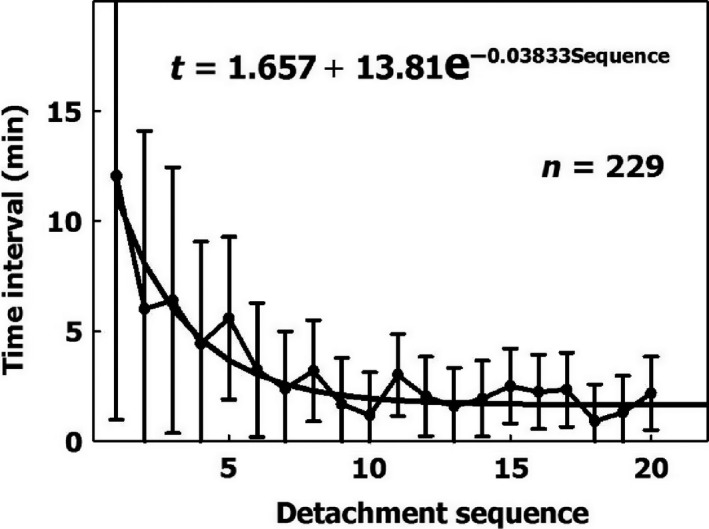
Time intervals (mean ± SD) between successive detachments from 24 active hydrophobic spots as a function of the detachment sequence. The first interval is the time from the end of decompression to the first detachment. The exponential equation was derived for the 229 data points.

As more bubbles become detached from an AHS, the AHS is activated and the rate of bubble production increases. After seven successive detachments, the time interval between further detachments stabilizes over a short period. This activation of the AHS explains why there are more bubbles in a second dive with the same profile on the same day (Dunford et al. 2002). Time intervals for the sequence of bubbles that become detached from an AHS are shown in Figure 8. The exponential equation which quantitates the activation was fitted with the actual data:

*t* = 1.657 + 13.81e^−0.03833×Sequence^ where *t* is the time interval in minutes between successive detachments and sequence is the number of bubbles which become detached from the same AHS. This equation can therefore be used for the increased risk due to prolonged decompression, a second dive on the same day, or subsequent daily dives (Dunford et al. 2002; Marinovic et al. 2009).

Variability between individuals, in the density and productivity of AHS, and in the activation of productive AHS, can all be used to design risk functions for decompression procedures.

### Age and acclimation

It has been suggested that the AHS are composed of lung surfactants deposited on the luminal aspect of blood vessels (Arieli 2015; Hills 1992). We recently confirmed the validity of this hypothesis, by showing that dipalmitoylphosphatidylcholine (DPPC) leaks from the lungs into the blood and settles to form AHS in the vessel (Arieli et al. 2016).

#### Age

In recent studies, advancing age was found to be a risk factor for DCI and decompression bubbles (Blatteau et al. 2011; Boussuges et al. 2009; Carturan et al. 2002). It was shown that this increased sensitivity is age‐ and not fat‐related (Schellart et al. 2012). We suggest that the number and area of AHS increase with age as a result of additional deposits of surfactants throughout life (Arieli 2015). This may explain the elevated risk of DCI and decompression bubbles with age. Therefore, an increase in the number and area of the AHS may be added to represent the risk due to age.

#### Acclimation

We also suggested that a detached bubble carries with it some of the phospholipids from the AHS (Arieli et al. 2015). Acclimation to diving, which can be seen in experienced divers who run less risk of DCI and produce low‐grade bubbles (Pontier et al. 2009a; Sayer et al. 2008; Zanchi et al. 2014), may be related to the depletion of phospholipids from the AHS.

The higher risk related to age, and the reduced risk related to frequent diving, may therefore be quantified according to the amount of DPPC in the AHS.

### Gas loading and unloading

Because bubble nucleation and expansion takes place mainly in the vascular compartment, the remaining tissues should be considered as inert gas sinks which are loaded with gas on compression, and on decompression, release this gas to the venous blood and the lungs. Therefore, the eight compartments previously suggested by Mapleson (1963) could be used to calculate mixed venous gas tension during the loading and unloading of inert gas: seven aqueous tissues (four visceral and three lean) having different perfusion/volume ratios, and one adipose tissue (Flook 2011). Diffusivity and solubility of gases in the tissues (assuming well mixed, perfusion‐limited compartments) should be considered for the different inert gases when a mixture of gases is used, and not the ratio of their diffusion coefficients in air as in Bühlmann (1984). Although diffusion limitation was demonstrated in the leg of a sheep, justifiable evidence of well‐mixed, perfusion‐limited compartments was shown for the brain and for the whole animal (D'Aoust and Lambertsen 1982; D'Aoust et al. 1977; Ohta and Farhi 1979).

### Individual modifications

It is also possible to adjust the model for a known decompression risk in a particular diver. A diver who has suffered more DCI than others should be placed in a high‐risk group. If PFO is known to exist, a higher risk should be scheduled. We recently showed that DPPC leaks from the lungs into the blood, settling at the AHS (Arieli et al. 2016). If the level of DPPC in plasma is found to correlate with sensitivity to DCI, a blood test will enable us to place a diver in the appropriate risk group.

## Conclusions

Because bubbles may be formed in the arterial circulation, we prefer the term DCI (illness) rather than DCS (sickness) to cover all symptoms of decompression‐induced disease. We believe that the vascular bubble model we propose adheres more closely to proven physiological processes and theory than do other suggested models. Its predictability may therefore be higher than other models, when appropriate adjustments are made for DCI data. Although the variability of the different parameters was derived from the sheep, whenever possible it does compare with diver variability and characteristics (Arieli et al. 2015). Vascular bubbles are the main and probably ultimate cause of all severe symptoms of DCI. In order to cover all symptoms, joint pain (“the bends”) will need to be modeled separately.

## Conflict of Interest

No conflicts of interest, financial or otherwise, are declared by the authors.

## Inert gas tension within an arterial segment

### Modeling diffusion during steady‐state arterial blood flow

V, Arterial blood flow; *r*, Inner radius; L, Length of the vessel;

W , Wall thickness (intima and media);

C0 – Inert gas concentration at the entrance to the vessel;

CT – Inert gas concentration in the tissue surrounding the vessel;

Cx – Inert gas concentration in the blood at distance x: both tissue and blood compartments are assumed to be well‐mixed;

D – Diffusion coefficient = 3.21 × 10^−9^ m^2^sec^−1^;

ΔC–Added inert gas concentration within the imaginary slice.

### Rate of entry and exit of the inert gas

VCx+2πrDΔX/W(CT‐CX)=V(Cx+ΔC)

ΔC/(CT‐Cx)=[(2πrD/WV)]ΔX

$$\int_{C0}^{Cx}\frac{\text{dC}}{CT-Cx}=\int_{0}^{x}\left(\frac{2\pi rD}{\text{WV}}\right)dx$$

$$\text{Cx}=\text{CT‐}\left(CT-C0\right)e^{(-2\pi D/\mathrm{WV})x}$$

### Concentration can be replaced by gas tension

$$\text{Px}=\text{PT‐}\left(PT-P0\right)e^{(-2\pi D/\mathrm{WV})x}$$
